# Nanotechnologies and Nanomaterials: Selected Papers from CCMR

**DOI:** 10.3390/nano14010036

**Published:** 2023-12-22

**Authors:** Ming-Yu Li, Jihoon Lee

**Affiliations:** 1 School of Science, Wuhan University of Technology, Wuhan 430070, China; 2 Department of Electronic Engineering, College of Electronics and Information, Kwangwoon University, Nowon-gu, Seoul 01897, Republic of Korea; jihoonleenano@gmail.com

Nanomaterial technology for the synthesis, processing, and fabrication of low-dimensional materials is where disciplines merge into a remarkable range of applications, from optoelectronics to health care (contribution 1–7), which affect the lives of millions. With the rapid development of science and technology, people have gradually realized that the variety of materials currently available cannot entirely meet the ever-increasing demand for minimalization and integration for devices. Nanomaterials, endowed with a facile controllability of directionally optical and electrical properties, have great potential to overcome the huge challenges related to the restricted area and balanced performance of multifunctional devices (contribution 8–10). Thus, the swift development of nanomaterial technology especially benefits burgeoning fields, such as Internet of Things [1], energy conversion [2], intelligent sensing [3], and even biomimetics [4].

Establishing how to efficiently control topology of nanomaterials is an essential issue in order to tailor their optical and electrical properties, which can be achieved by means of controllable growth in certain orientations, as well as stoichiometry. For chemical synthesis, the reaction of precursors is generally associated with catalyst and thermal supply, and isotropically spherical nanostructures can be obtained through individual nucleation and growth processes [5]. Meanwhile, anisotropic nanostructures (i.e., nanowires, nanorods, nanoflowers, etc.) are commonly synthesized via preferential growth in certain directions via selective passivation [6], and the obtained nanostructures can be uniformly dispersed into various solutions after ligand exchange. However, the long-term stability and well-aligned patterns of these nanomaterials still remain major problems for the practical applications of chemical synthesized nanostructures, and thus abundant research has focused on the synthesis methods [7], ligand exchange and passivation [8], and coating approaches [9], which will be briefly described in this Special Issue. For example, Henrieta Markevičiūtė et al. chemically deposited Ag–Se nanostructure films on a–Se/nylon, and the structural and optical properties of the composite nanostructures were systematically investigated (contribution 11). On the other hand, the nanostructures can also be directly fabricated via deposition and lithography, and the stoichiometry during deposition and the manufacturing cost are the two main concerns for the technique. In another study in this Special Issue, the fabrication approach for In-Sn-Zn oxide (ITZO) nanocomposite films via high-power impulse magnetron sputtering at room temperature is systematically investigated, and variations in carrier mobility can be correspondingly optimized through the a control of pulse off-time (contribution 12). To lower the expense for the lithography process, the nanopillar and nanohole arrays fabricated using the roll-to-roll nanoimprint lithography are systematically discussed in this Special Issue (contribution 13), which can uniformly realize large-scale manufacturing for well-defined nanostructures (contribution 14). In addition, various methods have been successfully realized to systematically engineer the surface morphology and stoichiometry for nanomaterials, i.e., electrostatic nano-assembly (contribution 15), pulsed-flow-induced fluidization (contribution 16), high-power-impulse magnetron sputtering (contribution 17), ink-jet printing (contribution 18), optimized e-beam-lithography (contribution 19), the simple template-free hydrothermal method (contribution 20), femtosecond laser-assisted fabrication (contribution 21), magnesiothermic catalysis (contribution 22), and even a simple grinding method (contribution 23). To date, the relatively facile approaches to achieve adjustable optical and electrical properties include variations to temperature (contribution 24,25), annealing durations (contribution 26), light radiation (contribution 27,28), and background gas (contribution 29,30). To accurately predict the effect of growth conditions, numerous simulation models and characterization systems have been established based on first principles studies (contribution 31–33) and micro-nano characterization techniques (contribution 34–36), respectively.

Photodetection can convert the message carried by light irradiation (i.e., wavelength, polarization, intensity) into readable electronic signals, and the performance of photodetectors strongly depends on photoactive layers. Nanomaterials with tunable optical properties depending on the topology provide a feasible approach for achieving an accurate response to light irradiation, triggering enthusiastic interest in developing sensitive low-dimensional photoactive materials. However, the disconnected morphology induced by many cracks during deposition is commonly believed to hinder carrier extraction and transfer within photoactive layers fabricated by nanomaterials, which urgently requires a plausible strategy to address. In another study in this Special Issue, ZnO quantum dots in a toluene solution are used as antisolvents for the formation of continuous films with 2D (PEA)_2_PbI_4_ nanosheets as a result of accelerated crystallization, and the carrier transfer highway is spontaneously established with the existence of ZnO quantum dots owing to the well-matched bandgaps (contribution 37). Collective oscillation of surface free electrons on metal nanoparticles can effectively concentrate incident light into photoactive layers, resulting in radically improved light absorption. Thus, silver triangular nanoprism arrays on WSe_2_ are proposed for fabricating spectra and polarization dual-sensitive photoreactors, which are realized by directionally boosted light absorption due to the silver triangular nanoprism arrays (contribution 38).

Additionally, the emission efficiency has been noticeably boosted by intensified hole injection (contribution 39) and spatial light modulation (contribution 40,41) with the existence of nanomaterials for light emitting and terahertz devices. In addition to light-emitting devices, nanomaterials have been widely witnessed in energy conversion devices and the synthesis of nanocomposites (contribution 42–44), and elaborately designed (contribution 45) nanostructures provide a facile approach for the enhancement of the conversion efficiency for Li-ion batteries and solar cells. Nanocomposites have been successfully introduced to electrochemical catalysts or photocatalysts during the hydrogen evolution (contribution 46–51) and oxygen evolution reactions (contribution 52–55), facilitating the carrier transition with semiconductor nanocomposites and even metallic materials. Nanotechnology has also emerged in the fabrication of transistors (contribution 56–59) and artificial synapses (contribution 60) for enhancing the performance with a controllable resistance for the barriers, and the conductivity of electrodes (contribution 61) and capacity (contribution 62) for supercapacitors can be also effectively optimized via nanotechnologies.

This Special Issue also includes studies on the novel strategies for distinctive sensing techniques, including chemical sensors, gas monitoring, biosensors, acoustic sensors, and even special detection for different irradiations (contribution 63–7 6). We sincerely hope readers will find useful information for their research, and that all of these works can spark more interesting ideas in various research fields.
